# Does automatic tube compensation change metabolic parameters when added to pressure support mode during weaning?

**DOI:** 10.1186/2197-425X-3-S1-A321

**Published:** 2015-10-01

**Authors:** I Karaca, A Comert, A Kuntman, K Demirag, M Uyar

**Affiliations:** Ege University Hospital, Izmir, Turkey

## Introduction

Artificial airway increases work of breathing (WOB) during weaning [1] which is one of the reasons for weaning failure [2]. Pressure support ventilation (PSV) decreases WOB and oxygen expenditure by decreasing resistance caused by artificial airway. Because inspiratory flow changes with every breath during PSV, it can not constantly compansate the changes in resistance caused by changes due to patient's inspiratory effort.

Automatic tube compensation (ATC) compensates the imposed WOB due to artificial airway. ATC adjusts the pressure inside the endotracheal tube therefore we hypothesized that when ATC is added to PSV, this compensation becomes better.

## Objectives

To investigate the effects of addition of ATC to PSV on metabolic parameters.

## Methods

After approval from University Ethics Committee and having patients´ informed consents, patients ventilated with PSV mode more than 24 hours and met the weaning criteria were included. Patients having a respiratory rate>35/min, dyssynchrony with ventilator, body temperature>38.3oC, abnormal blood gas values, hemodynamic unstability were discarded. After obtaining normal blood gas values at the end of a stabilization period with PSV, metabolic parameters were measured for 30 min and average values of oxygen consumption (V02), carbondioxide production (VC02) and energy expenditure (EE) were recorded. Then ATC set at 100% support was added to PSV and after the similar adaptation period, measurements were repeated for 30 min. Vital signs, respiratory mechanics and blood gas results were recorded at every 10 min.

During the study period of 2 hours, patients were not disturbed by tracheal aspiration, position change and wound and catheter dressings. If needed, patient was discarded.

## Results

Thirty-nine patients with an average age of 54,8 ± 21,5 years, APACHE II score of 20,7 ± 6,5 and ideal body weight of 64,4 ± 9,07 kg were enrolled. There were no significant difference in terms of hemodynamic parameters, blood gas values and respiratory mechanics between PSV alone and PSV + ATC. Metabolic measurements were also not different (Table 1).

**Table 1:** Metabolic measurements

	PSV	PSV + ATC	p
Energy expenditure (kcal)	1728,2 ± 430,5	1696,9 ± 418,3	0,195
Oxygen consumption (ml/min)	259,3 ± 68,7	251,3 ± 65,4	0,129
Carbondioxide production (ml/min)	201,0 ± 55,5	199,58 ± 52,2	0,646

## Conclusions

The addition of ATC to a standart PSV based weaning did not improve metabolic parameters. More clinical studies are needed to clarify the effect of ATC as an adjunct measure during weaning.
